# Pv3Rs: *Plasmodium vivax* relapse, recrudescence, and reinfection statistical genetic inference

**DOI:** 10.1093/bioinformatics/btaf643

**Published:** 2025-12-01

**Authors:** Yong See Foo, Michael T White, Aimee R Taylor

**Affiliations:** School of Mathematics and Statistics, University of Melbourne, Parkville, VIC 3010, Australia; Department of Global Health, Institut Pasteur, Université Paris Cité, Paris 75015, France; Department of Global Health, Institut Pasteur, Université Paris Cité, Paris 75015, France

## Abstract

**Summary:**

A *Plasmodium vivax* recurrence can be caused by activation of dormant liver-stage hypnozoites (relapse), failure to successfully treat a blood-stage infection (recrudescence), or a new mosquito inoculation (reinfection) (Pv3Rs). Inference of the cause of recurrence is important, especially in clinical trials, where molecular correction is used to improve estimates of antimalarial treatment efficacy. Pv3Rs implements statistical inference of *P. vivax* recurrence states (recrudescence, relapse, reinfection) from *P. vivax* genetic data. Under various simplifying assumptions, we model genetic relationships between parasites within and across infections to compute the posterior probabilities of recurrence states. Pv3Rs is applicable to a variety of genetic data types, including length polymorphic loci and amplicon sequencing data.

**Availability and implementation:**

Pv3Rs is freely available on CRAN under the GNU license. Its CRAN DOI is 10.32614/CRAN.package.Pv3Rs. Source code and installation instructions can be found at https://cran.r-project.org/web/packages/Pv3Rs/index.html.

## 1 Introduction

Among the species of *Plasmodium* parasites that cause malaria in humans, *Plasmodium vivax* is considered the most difficult to eliminate, in large part because of its ability to relapse. Relapses are blood-stage infections derived from the activation of liver-stage parasites called hypnozoites (Markus 2019); hypnozoites can remain dormant in the liver (the first human stage of the malaria parasite life cycle following inoculation) for more than a year. A *P. vivax* recurrence can also be caused by failure to successfully treat a previous blood-stage infection (recrudescence), or—in endemic settings—by a new infectious mosquito bite (reinfection). Identifying the cause of *P. vivax* recurrence is important, especially in clinical trials where identification improves efficacy estimation. For example, efficacy estimates of radical cure (treatment of both liver- and blood-stage parasites) would be downwardly biased if reinfections were mistaken for relapse or recrudescence. The cause of recurrent *Plasmodium falciparum* malaria is typically resolved via molecular correction, that is, the use of *P. falciparum* genetic data to distinguish between recrudescence and reinfection (*P. falciparum* does not relapse) (Hastings and Felger 2022). Because relapses complicate matters, there is no standard approach to *P. vivax* molecular correction at present.

In response, we have built an R package Pv3Rs to perform statistical genetic inference for the probabilities of *P. vivax*  recrudescence, relapse, and reinfection (Pv3Rs). Our approach leverages the fact that each cause of recurrence is associated with a different profile of genetic relationships between parasites across infections. Upon the specification of observed data and allele frequencies, Pv3Rs can be used to compute and visualize the posterior probabilities of the causes of recurrence under a Bayesian hierarchical model that makes various simplifying assumptions. The package also includes visualization utilities for the observed data and allele frequencies.

## 2 Methods

Consider a trial participant with an enrolment episode of *P. vivax* malaria (episode 0) and *k* recurrent episodes of *P. vivax* malaria (episodes 1,…,k), for which we have *P. vivax* genetic data over *m* markers from blood samples obtained. Let $y_{tj}$ denote the alleles detected at marker j=1,…,m during episode t=0,…,k. The cause of each recurrent episode t=1,…k, denoted as $s_{t}$, is either recrudescence (C), relapse (L), or reinfection (I). We refer to these possible causes as recurrence states, and model them as mutually exclusive. The main objective of Pv3Rs is to compute the posterior probability of each possible sequence of recurrence states $s=(s_{1},\ldots ,s_{k})$ conditional on the genetic data y (the trial participant’s collection of all $y_{tj}$). Apart from genetic data, posterior computation also requires the user to provide, for each marker *j*, the allele frequencies $f_{j}$.

A simple Pv3Rs workflow consists of three steps:

1. plot_data to visualize the genetic data y, possibly along with the allele frequencies $f=(f_{1},\ldots ,f_{m})$
2. compute_posterior to compute, for each state sequence s, the posterior probability P(s|y)
3. plot_simplex to visualize per-recurrence posterior probabilities of recrudescence, relapse, and reinfection

These steps are illustrated in the function documentation using a running example based on real data.

### 2.1 Data visualization

**Figure 1.**
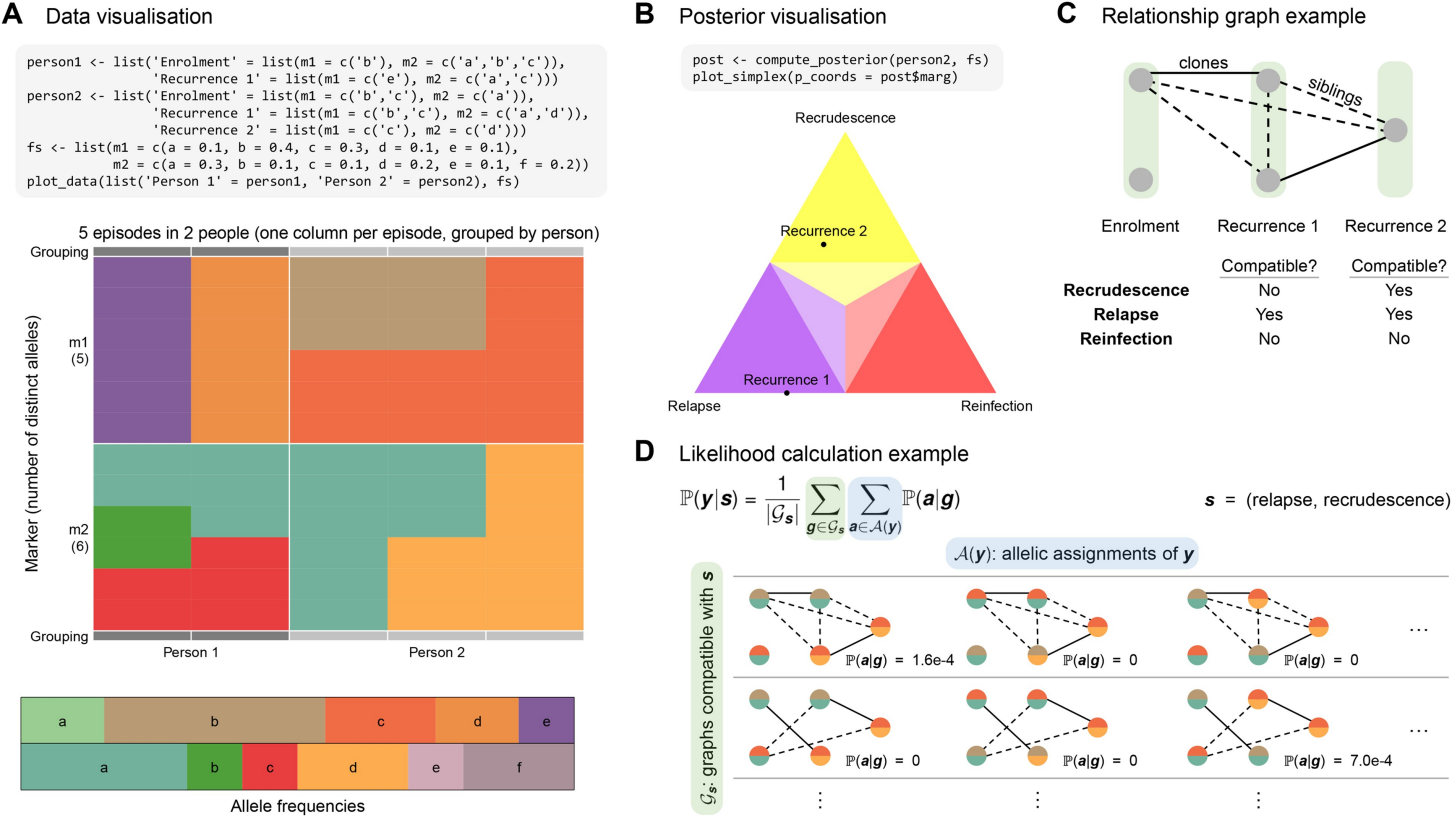
(A) Visualization of genetic data of two participants and allele frequencies using plot_data. Cells are coloured according to the corresponding alleles. There are two rows corresponding to two markers (m1 and m2), which are further split into a variable number of cells, reflecting the number of alleles observed for each episode at that marker. Columns correspond to episodes in chronological order (left to right), and are grouped by participant (grey bars). (B) Visualization of per-recurrence posterior probabilities for the second participant using plot_simplex. The closer a point is to a corner, the more likely the corresponding recurrence state; a point on an edge indicates zero probability for the state corresponding to the opposite corner. (C) An example of a relationship graph for the second participant. Clones are linked by solid edges, siblings are linked by dashed edges, strangers are unlinked. (D) Likelihood calculation of a recurrence state sequence s, illustrated using the second participant. Rows correspond to relationship graphs $g\in G_{s}$ that are compatible with s. Columns correspond to allelic assignments a∈A(y) (ways to assign allelic data y to graph vertices). The probability of an allelic assignment a given a relationship graph g, P(a|s), is zero if parasite genotypes connected by a clonal edge have different alleles.

In Fig. 1A, we use the function plot_data to visualize synthetic genetic data for two people. Episodes are ordered chronologically from left to right, grouped by person. The grey bars along the top and bottom edges indicate that, in addition to an enrolment episode, Person 1 has one recurrent episode, whereas Person 2 has two recurrent episodes. Note that during one episode, there may be multiple alleles detected at one marker, e.g. during the enrolment episode of Person 1, there are thee alleles detected at marker m2. Accompanying the table of detected alleles are colour bars depicting the allele frequencies f, which share the same vertical ordering for markers. This visualization functionality is not limited to *P. vivax* data: it can be used to visualize *P. falciparum* genetic data intended for molecular correction of routine antimalarial therapeutic efficacy studies.

### 2.2 Statistical inference

At the core of Pv3Rs is the statistical inference of recurrence states, which the function compute_posterior implements by summing over all latent variables of the model described in Section 3. Given genetic data on one person y and allele frequencies f, compute_posterior returns the posterior distribution P(s|y). Recall that s is a sequence of *k* recurrence states, where *k* is the number of recurrences and each state is one of {C,L,I}. The distribution P(s|y) consists of $3^{k}$ probabilities corresponding to each possible state sequence. In practice, we may be interested in the state probabilities of individual recurrences, instead of the full sequence. These are known as marginal posterior probabilities $P(s_{t}|y)$ for each recurrence *t*. For example, the probability that the first recurrence is a recrudescence, $P(s_{1}=C|y)$, is the sum of probabilities P(s|y) taken over the $3^{k-1}$ sequences s satisfying $s_{1}=C$. The function compute_posterior returns a list. The list component named joint is a vector of the $3^{k}$ state sequence probabilities; the list component named marg is a k×3 matrix of per-recurrence state probabilities.

Formally, the posterior distribution is defined as follows:


(1) $$P(s|y)=\frac{P(y|s)P(s)}{\sum_{s\in {\left\{C,L,I\right\}}^{k}}P(y|s)P(s)},$$

where P(y|s) is the probability of observing y if the state sequence of an individual is s (likelihood), and P(s) is a distribution over all possible state sequences encoding our belief about s in the absence of genetic data (prior). We defer the explanation of the likelihood calculation to Section 3. The user may choose to specify the prior distribution (an externally generated matrix of per-recurrence recurrence state probabilities) as informed by additional data sources, such as time-to-event data, which can be very informative (White 2011, Battle *et al.* 2014), and were used by Taylor *et al.* (2019), via the prior argument to compute_posterior. We recommended using additional information where available. The default prior consists of independent uniform priors over $s_{t}\in \{C,L,I\}$ for each recurrence t=1,…,k, i.e. $P(s_{t})=1/3$. This choice is made to highlight the lack of alternative information; there is no epidemiological support for it.

### 2.3 Posterior visualization

Per-recurrence probabilities of recrudescence, relapse, and reinfection (i.e. marginal state probabilities) can be visualized on a simplex plot, via the function plot_simplex. We illustrate this in Fig. 1B, using Person 2 from Fig. 1A as an example. The visualization shows that the first recurrence is most likely to be a relapse, whereas the second recurrence is most likely to be a recrudescence. The closer a point is to a corner, the higher the probability of the corresponding state (at the centre of the triangle, all three states are equally likely). Colours indicate which state is most likely, e.g. purple for relapse. Regions with a darker shading indicate that the corresponding probability is >0.5.

## 3 Statistical model and assumptions

Recrudescence, relapse, and reinfection draw parasites derived from different sources. As such, they generate parasites with different genetic relationship profiles. These relationships are the result of sexual recombination between parental gametes in a mosquito blood meal (Fig. 1, available as supplementary data at *Bioinformatics* online). Recrudescent parasites are asexual replicates of parasites from a previous blood-stage infection, thus recrudescence generates clones (barring *de novo* mutations). Reinfection draws parasites from a new mosquito inoculation, thus a different blood meal; we call these parasites strangers. They may be related insofar as the parasite population is inbred and structured. Relapsing parasites are a delayed draw from one or more past mosquito inoculations. Thus, relapse can generate clones, strangers, and siblings, which can be meiotic, parent–child-like, regular and half siblings; see Fig. 1, available as supplementary data at *Bioinformatics* online.

The Pv3Rs model is built around a simplified version of these relationships; a detailed description of the model and its assumptions is available in the Supplementary Material. We model relationships between parasite genotypes via relationship graphs: graphs whose vertices (parasite genotypes) are unlinked in the case of strangers, or linked by either clonal or sibling edges (Fig. 1C). Since observed allelic data y are not experimentally assigned to parasite genotypes, we need to consider all the ways to assign alleles to vertices (Fig. 1D); this is not unique for polyclonal episodes.

To define the likelihood of a state sequence s, P(y|s), we need to prescribe a distribution over the set of graphs compatible with s, denoted as $G_{s}$. Following Taylor *et al.* (2019), we assume that all graphs in $G_{s}$ are equally likely, thus P(y|s) is the average of graph likelihoods P(y|g) over all graphs in $G_{s}$ (see equation in Fig. 1D). The likelihood of a graph g, P(y|g), is computed by summing over all allelic assignments. In general, the number of allelic assignments is exponential in the number of markers, but a decomposition trick allows the likelihood to be computed in linear time (Section 2.3, available as supplementary data at *Bioinformatics* online). The probability of an allelic assignment given a graph, P(a|g), is computed by summing over marker-wise clusters of identity-by-descent, where each cluster draws a single allele with probability equal to its frequency (Section 2.2, available as supplementary data at *Bioinformatics* online).

Some of Pv3Rs’ internal computations can be exposed to the user via optional arguments to compute_posterior, namely return. RG and return.logp. When return. RG is set to TRUE, all valid relationship graphs are returned, which can be visualized using the function plot_RG. When both return. RG and return.logp are set to TRUE, the log likelihood of each relationship graph is returned. Our relationship graph model is based on various simplifying assumptions, such as treating all siblings as regular siblings, assuming that recrudescent parasites are derived only from the directly preceding episode, and that recrudescence, relapse, and reinfection are mutually exclusive. Moreover, we do not model undetected alleles, genotyping errors, *de novo* mutations, or parasite population structure. These assumptions result in various limitations discussed below.

## 4 Discussion

### 4.1 Pv3Rs in context

Pv3Rs is the only user-friendly tool for *P. vivax* molecular correction. For *P. falciparum* molecular correction, the World Health Organization (WHO) recommends match-counting algorithms (WHO 2008, 2021). There are no WHO-endorsed match-counting algorithms for *P. vivax* because relapsing parasites complicate classification. If the WHO-endorsed *P. falciparum* match-counting algorithms were applied to *P. vivax*, each relapse would be misclassified as either a recrudescence or a reinfection in a setting-dependent manner. For example, reinfection misclassification would likely dominate in a high transmission setting where people accumulate banks of genetically diverse hypnozoites; meanwhile, recrudescence misclassification could dominate in a low transmission setting where clonal propagation, inbred siblings, and identity-by-chance are common. Consequently, most studies that analyse data on *P. vivax* recurrences use heuristic approaches, including bespoke match-counting, and classification based on inter-episode relatedness estimates. One exception is the study done using the Pv3Rs’ prototype (Taylor *et al.* 2019). It was the first demonstration that statistical genetic inference of *P. vivax* recurrence is feasible; the prototype was implemented as open-source but study-specific scripts, not a user-friendly R package.

Pv3Rs’ model is computationally more powerful than its prototype (Taylor *et al.* 2019): it supports data on hundreds of markers, and it is not limited to only two recurrences per person (Section 3.1, available as supplementary data at *Bioinformatics* online). Moreover, correction of a mathematically convenient but biologically meaningless assumption by Taylor *et al.* (2019) (Section 3.2, available as supplementary data at *Bioinformatics* online) entails a strict assumption that makes statistical inference using Pv3Rs more liable to half-sibling misspecification.

### 4.2 Limitations and future work

Pv3Rs generates posterior probabilities under a Bayesian framework, which are conditional on both the *data* and the *model*; the validity of our output depends on how well our model describes the true data-generating process. We discuss some of our model’s limitations below, and their interactions with transmission intensity in Section 3.5, available as supplementary data at *Bioinformatics* online.

Computation remains limited to per-participant data whose genotype count is moderate (ideally fewer than nine; not more than 10). If a total genotype counts exceeding eight is distributed over multiple recurrences, it might be possible to compute posterior probabilities by analysing episodes pairwise [this approach was used by Taylor *et al.* (2019)].

We model all sibling parasites as regular siblings, e.g. drawing from at most two parental alleles [not true of half siblings, or of Taylor *et al.* (2019)]. In our experience, half-sibling misspecification sometimes leads to some misclassification of relapses as reinfections (Section 3.3, available as supplementary data at *Bioinformatics* online). Development of a simple diagnostic that can be used to identify instances of half-sibling misspecification is merited.

We do not model undetected alleles, genotyping errors, or *de novo* mutations. Recall that recrudescent parasites are modelled as perfect clones under Pv3Rs. As such, the posterior probability of recrudescence is rendered zero by errors and mutations. This becomes more likely when there are data on more markers. In addition, false positive alleles may generate genotype counts that exceed the aforementioned computational limit. A preliminary analysis on the impact of errors and mutations is discussed in Section 3.5, available as supplementary data at *Bioinformatics* online.

Pv3Rs was designed with a view towards clinical trials where study participants are actively followed up frequently and where all detected infections are treated to the extent that post-treatment parasitaemia drops below some detectable level before recurrence, if recurrence occurs. In studies where infections come and go undetected, persist untreated, and/or where events have time to accumulate, the concept of recurrence is ill-defined, and the output of Pv3Rs might not be meaningful.

We do not model all the complexities that can confound molecular correction. For example, we do not model any population structure (e.g. household effects) or the failure to capture a low-density clone in the body by a blood sample of limited volume (Snounou and Beck 1998), nor hidden biomass in the spleen and bone marrow (an alternative source of *P. vivax* recurrence) (Markus 2019). Users should interpret probabilities conditional on the model and how it relates to the study design and the methods used to generate data. For example, we expect Pv3Rs to output probable relapse if a person is reinfected by a new mosquito with parasites that are recently related to those that caused a previous infection, as might happen in household transmission chains. Randomization mitigates the ramifications of some of these issues in multi-arm trails.

Because clonal parasites can be generated by either recrudescence or relapse, and stranger parasites can be generated by either reinfection or relapse, there is always some posterior probability of relapse, unless relapse is discounted *a priori*. Users should interpret recrudescence and reinfection posterior probabilities as probabilities that are differentially constrained across participants: different parasite genotype counts induce different posterior bounds because inference depends on graphs whose vertices are parasite genotypes. Some of these bounds can be derived *a priori* due to the uniform assumption on graphs (Section 3.4, available as supplementary data at *Bioinformatics* online). For example, under the default model, we can be at most 75% certain of recrudescence for a participant with a single parasite genotype at enrolment and recurrence. If the uniform assumption on graphs is violated, residual relapse probabilities may be downwardly biased. For example, if a participant is more likely to relapse with strangers than clones because they have amassed a large bank of genetically diverse hypnozoites, the residual relapse probability given probable reinfection is likely biased downwards. Meanwhile, if a participant’s hypnozoites all derive from a single mosquito whose sporozoites were monoclonal, the residual relapse probability given probable recrudescence is likely biased downwards. The development of a more biologically principled generative model on parasite relationships is merited.

## 5 Conclusion

Pv3Rs is the first user-friendly software for *P. vivax* molecular correction. We anticipate Pv3Rs will help standardize analyses of clinical trial data for improved antimalarial efficacy estimation.

## Supplementary Material

btaf643_Supplementary_Data
