# Supplementary material for: Pv3Rs: Plasmodium vivax relapse, recrudescence, and reinfection statistical genetic inference
Source: Bioinformatics. 2025 Dec 1;42(1):btaf643. doi: 10.1093/bioinformatics/btaf643 (PMC12758599; doi:10.1093/bioinformatics/btaf643)
Supplement: btaf643_Supplementary_Data [file btaf643_supplementary_data.pdf]

Supplement of Pv3Rs: *Plasmodium vivax* relapse, recrudescence, and reinfection statistical genetic inference

Yong See Foo, Michael T White, Aimee R Taylor

This supplement is adapted from the preprint Taylor et al. (2022).

Contents

1 Background **1**

2 Model **4**

2.1 Overview 4

2.2 Mathematical description 5

2.3 Decomposing the phasing space 6

3 Additional details **8**

3.1 Computational scaling 9

3.2 Deviation of Pv3Rs model assumptions from prototype 9

3.3 Half-sibling misspecification 10

3.4 Influence of uniform distribution over graphs 10

3.5 Dealing with misspecification 11

1 Background

We use the following attributes of *P. vivax* biology and epidemiology to build a statistical genetic model of recurrent vivax malaria.

P. vivax parasites are haploid in the human stage of their life-cycle, but they can reside in infections with multiplicities of infection (MOIs) greater than one (i.e., there can be more than one genetically distinct haploid genotype per infection). Soon after being ingested by a mosquito, pairs of *P. vivax* gametocytes (human-to-mosquito transmission stage) transform into gametes and recombine sexually, with one pair of gametes (i.e., a zygote) per oocyst, one or more oocysts per mosquito, and possible selfing (recombination between genetically identical gametes). Each zygote generates four haploid meiotic offspring.[1] Each offspring then replicates asexually, generating thousands of sporozoites (mosquito-to-human infectious stage) that hatch out of the oocyst/s and migrate to the salivary glands of the mosquito (Smith and Barillas-Mury, 2016). The Pv3Rs model is built partly around the consequent fact that a parasite brood (collection of parasites produced when one or more oocysts derived from the same blood meal hatch) can contain parasites that are clones, siblings and strangers (Figure S1), where relationships are defined meticulously for our specific purpose (Table S1), and that all derived parasites in the human stage of the life-cycle are asexual replicates.

A single mosquito inoculation can can contain parasites from different broods if the mosquito has fed on infectious blood meals more than approximately one hour apart (Smith and Barillas-Mury, 2016). Sporozoites from different broods, and thus different mosquitoes, are strangers. They are related insofar as the population across infected humans is inbred and structured (typically, mosquitoes take blood meals days apart (Scott and Takken, 2012), and thus on different people, albeit with some exception, especially among people from the same household (Koella et al., 1998)).

The Pv3Rs model is structured around three processes that each generate recurrent blood-stage *P. vivax* parasites: recrudescence generates blood-stage parasites that are asexual replicates of those in the directly preceding blood-stage infection; reinfection generates blood-stage parasites derived from a new inoculation, relapse generates blood-stage parasites derived from one or more previous inoculations (Figure S2). As such, the 3Rs can be reframed in terms of generating parasites that are strangers, clones, and siblings of parasites in

[1] **Meiotic offspring do not draw alleles from parental chromatids with replacement:** If an offspring has drawn an allele from one chromatid of the first parent, another offspring has a ⅓ chance of drawing from the remaining first-parent chromatid, and a ⅔ chance of drawing from a second-parent chromatid; meiotic siblings whose parents are unrelated are thus 33% related on average, cf. regular siblings, which are draws from different oocysts and thus independent meiotic events)

preceding blood-stage infection/s: recrudescence generates clones of the parasites in the preceding blood-stage infection; reinfection generates strangers of parasites in preceding blood-stage infections; relapse can generate clones, strangers, and/or siblings of parasites in preceding blood-stage infections. In addition to strangers within a single mosquito inoculation, relapses can generate strangers because study participants in endemic settings are liable to accumulate a live bank of genetically diverse hypnozoites from antecedent infections (Figure S2).

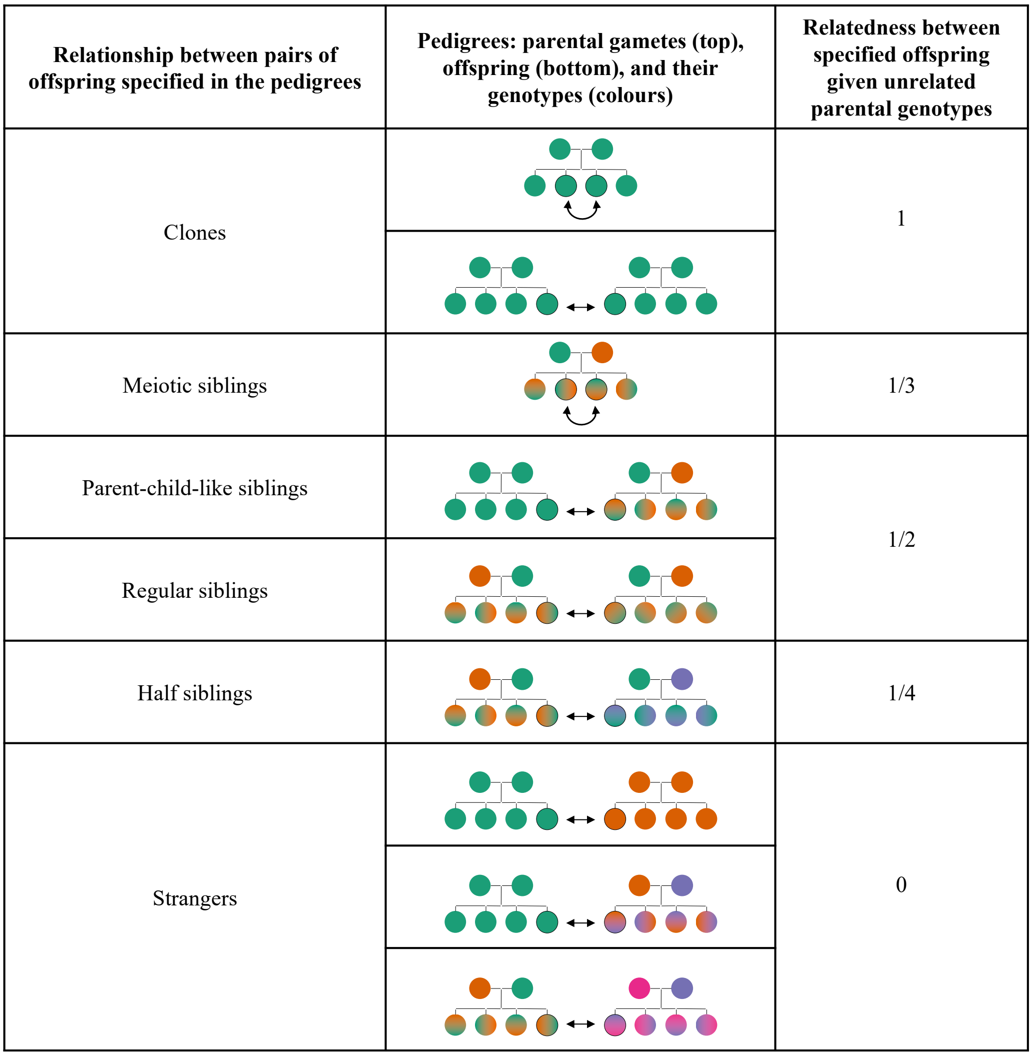

Figure S1: Intra-brood relationships: among the four parental gametes from which a pair of sporozoites that belong to the same brood are ultimately derived, there is at most four parental genotypes (represented here by solid green, orange, purple and pink) leading to a limited set of inter-offspring (and thus inter-sporozoite) relationships within a parasite brood. Although siblings have in common at least one parental genotype, they are distinct (represented by different orientations of colour gradients). This figure is an adaptation of Figure 2 of (Wong et al., 2018).

**Episode**	A detectable blood-stage infection.
**Recurrence**	A malaria episode following an episode-free time interval preceded by an episode.
**Relapse**	Recurrence with parasites derived from the activation of a hypnozoite.
**Reinfection**	Recurrence with parasites dervied from a new infectious mosquito bite.
**Recrudescence**	Recurrence with parasites derived from those in a preceding episode; i.e., failure to fully treat the preceding blood-stage infection.
**Brood**	Collection of parasites produced when oocysts derived from the same blood meal hatch; i.e., offspring derived from parental gametes imbibed at the same time.
**Clones**	Parasites derived from the same brood that share the same genotype (deviates from the common definition of a clone due to the same-brood condition).
**Siblings**	Parasites derived from the same brood that have different genotypes but have in common one or two parental genotypes. Sibling parasites can either be derived from the same zygote (meitoic siblings) or from different zygotes. Those from different zygotes can have in common one parental genotype among two (siblings that resemble parent-child pairs), two parental genotypes among two (regular siblings), or one parental genotype among three (half siblings).
**Strangers**	Parasites derived from the same brood that do not have a common parental genotype and parasites derived from different broods.
**Realistic population**	Strangers are related insofar as the population across infected humans is inbred and structured (e.g., over households). In an extreme scenario without population diversity, stranger parasites are 100% related (common definition of a clone) and genetic data are uninformative. Intra-brood stranger parasites and sibling parasites are liable to have elevated relatedness if their parental parasites were obtained via mosquito-to-human cotransmission.
**Idealised population**	Strangers are unrelated and siblings are either ¼ related (half siblings), ⅓ related (mieotic siblings), or ½ related (regular and parent-child-like siblings).
**Genotype**	A specific realisation of the haploid parasite genome.
**Multiplicity of infection**	A per-infection count of the number of distinct genotypes.

Table S1: Working definitions; some differ to those used commonly in the literature (e.g., see clone).

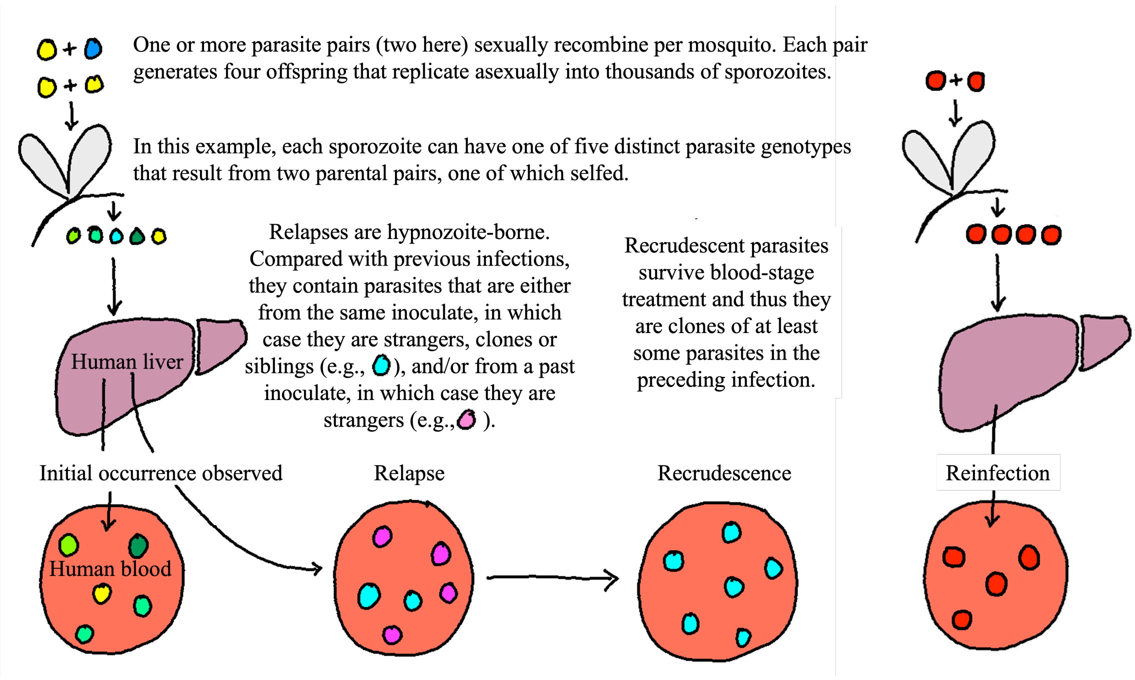

Figure S2: Recurrent vivax malaria. Different colours represent genetically distinct haploid genotypes. Green parasites are derived from the offspring of the cross between yellow and blue parasites.

2 Model

2.1 Overview

Under the model we assume clones have relatedness one, i.e., we ignore *de novo* mutations and ectopic recombination between *var* genes etc. We assume both strangers and the parental genotypes of siblings are unrelated.

Having redefined the recurrence states (recrudescence, relapse, reinfection) in terms of processes that generate clones, strangers and siblings, we compute the probability of a recurrence state conditional on all data for a given study participant by summing over latent graphs of clonal, sibling and stranger relationships between genotypes within and across infections. We assume a uniform prior distribution over graphs compatible with a given recurrence state: all graphs are compatible with relapses; all graphs with clonal edges connecting genotypes in the specified recurrence to genotypes in the directly preceding infection are compatible with recrudescence; all graphs with only stranger edges connecting genotypes in the specified recurrence to genotypes in all preceding infections are compatible with reinfection.

We model observed allelic data assuming perfect detection, no error and no mutation. Since no attempt is made to model genotyping error, the model is agnostic to marker type. Although we do not model error between observed and latent alleles, we do make a distinction between observed allelic data (unphased) and unobserved genotypes (vectors of phased alleles). A comprehensive set of model assumptions is listed below.

1. Perfect detection of alleles
2. No genotyping errors
3. No *de novo* mutations
4. Missing data are ignorable
5. Markers are conditionally independent and neutral
6. All siblings are regular siblings: they draw parental alleles with replacement (not true of meiotic siblings) from at most two parents (half siblings draw from three parents) and are transitive (not true of parent-child-like sibling trios or some half sibling trios)[2]
7. The parasite population is unstructured. As such, the parental genotypes of siblings and strangers are related only insofar as allele frequencies encode population-averaged relatedness marker-by-marker (see Mehra et al. (2025)). In reality, parental genotypes and strangers that are co-transmitted are likely related; among parental genotypes and strangers from different mosquitoes, those from mosquitoes close together in space and time are more likely related than those from mosquitoes far apart in space and time.
8. Mutually exclusive recurrence states: requires frequent and active follow-up with treatment that reduces parasitaemia below the limit detection; incompatible with study designs where blood-stage infections persist untreated.
9. The MOI of recrudescent infection is less than or equal to the MOI of the preceding infection (follows from assumptions 1, 2, 3 and 8), and a recrudescence is defined relative to the directly preceding infection only.

[2]**Sibling relationships need not be transitive:** Under the Pv3Rs models, sibling relationships are always transitive (the distribution over graphs is a distribution over transitive graphs). However, a mosquito with an MOI of two could have three oocysts: one selfed with one genotype, another selfed with the other genotype, and one cross. If a single sporozoite is sampled from each oocyst, the trio is not transitive. Half-sibling trios are not necessarily transitive neither (e.g., a trio of crosses between parental genotypes AB, BC, AC is transitive, but a trio of crosses between genotypes AB, BC, CD is not transitive). If we dropped sibling transitivity altogether, the number of relationship graphs would be

$$\sum_{k=1}^{n} B(n,k)x^{\binom{k}{2}},$$

where n is the number of genotypes (not necessarily unique), $B(n,k)$ is the number of ways of partitioning n genotypes into k clonal groups, x is the number of types of relationships between clonal groups (two if only sibling or stranger; three if half-sibling, sibling or stranger), and $\binom{k}{2}$ is the number of edges between clonal groups. Unfortunately, dropping transitivity altogether generates invalid graphs, e.g., it would generate graphs with two regular sibling edges plus one stranger edge.

2.2 Mathematical description

The Pv3Rs model described mathematically below, is depicted graphically in Figure S3. A summary of notation is provided in Table S2.

Let k denote some number of recurrent vivax malaria episodes experienced by an individual. Suppose $\boldsymbol{y} = (\boldsymbol{y}_{tj})$ is a matrix of *P. vivax* genetic data. Specifically, a matrix of sets, where each set, $\boldsymbol{y}_{tj}$, contains the alleles observed in the $t \in 0, \dots, k$ episode at the jth marker genotyped. Conditional on $\boldsymbol{y}$, Pv3Rs computes the posterior probability that the state of the tth recurrence, s_t, is a recrudescence C, relapse, L, or reinfection, I. It does so by summing over all sequences of recurrence states, $\boldsymbol{s} = (s_1, \dots, s_k)$, in the sequence space $\boldsymbol{\mathcal{S}}$ where $s_t = C$, L or I, respectively,

$$\text{e.g.,} \quad \mathbb{P}(s_t = L|\boldsymbol{y}) = \sum_{\boldsymbol{s} \in \boldsymbol{\mathcal{S}}: s_t = L} \mathbb{P}(\boldsymbol{s}|\boldsymbol{y}).$$

The posterior probability, $\mathbb{P}(\boldsymbol{s}|\boldsymbol{y})$, is computed assuming that recurrence states are *a priori* independent across episodes, ideally using informative priors (e.g., setting $\mathbb{P}(s_t)$ equal to the output of a time-to-event model as in (Taylor et al., 2019),

$$\mathbb{P}(\boldsymbol{s}|\boldsymbol{y}) = \frac{\mathbb{P}(\boldsymbol{y}|\boldsymbol{s})\mathbb{P}(\boldsymbol{s})}{\mathbb{P}(\boldsymbol{y})}, \quad \mathbb{P}(\boldsymbol{y}) = \sum_{\boldsymbol{s} \in \boldsymbol{\mathcal{S}}} \mathbb{P}(\boldsymbol{y}|\boldsymbol{s})\mathbb{P}(\boldsymbol{s}), \quad \mathbb{P}(\boldsymbol{s}) = \prod_{t=1}^{k} \mathbb{P}(s_t).$$

If one wishes to use a prior distribution $\mathbb{P}_{\text{dep}}(\boldsymbol{s})$ with dependence across episodes, this desired posterior distribution can be obtained with a post-processing step on posterior probabilities obtained using a uniform prior. The posterior probabilities obtained using a uniform prior satisfy

$$\mathbb{P}_{\text{unif}}(\boldsymbol{s}|\boldsymbol{y}) \propto \mathbb{P}(\boldsymbol{y}|\boldsymbol{s}),$$

thus the posterior probabilities based on the episode-dependent prior $\mathbb{P}_{\text{dep}}(\boldsymbol{s})$ can be computed as

$$\mathbb{P}_{\text{dep}}(\boldsymbol{s}|\boldsymbol{y}) = \frac{\mathbb{P}(\boldsymbol{y}|\boldsymbol{s})\mathbb{P}_{\text{dep}}(\boldsymbol{s})}{\sum_{\boldsymbol{s} \in \boldsymbol{\mathcal{S}}} \mathbb{P}(\boldsymbol{y}|\boldsymbol{s})\mathbb{P}_{\text{dep}}(\boldsymbol{s})} = \frac{\mathbb{P}_{\text{unif}}(\boldsymbol{s}|\boldsymbol{y})\mathbb{P}_{\text{dep}}(\boldsymbol{s})}{\sum_{\boldsymbol{s} \in \boldsymbol{\mathcal{S}}} \mathbb{P}_{\text{unif}}(\boldsymbol{s}|\boldsymbol{y})\mathbb{P}_{\text{dep}}(\boldsymbol{s})}.$$

The likelihood, $\mathbb{P}(\boldsymbol{y}|\boldsymbol{s})$, sums over various latent variables. Because relationships are genotype-level attributes, in the description that follows, we find it more intuitive to first phase the data into genotypes and then sum over relationship graphs between genotypes. More specifically, we first sum over every matrix of phased alleles, $\boldsymbol{a} = (a_{ij})$, in matrix space $\boldsymbol{\mathcal{A}}$ (equation (1)). Each $\boldsymbol{a} \in \boldsymbol{\mathcal{A}}$ represents one way to phase the observed alleles into $i = 1, \dots, n$ vectors of $j = 1, \dots, m$ alleles. Each vector, $\boldsymbol{a}_{i\cdot} = (a_{i1}, \dots, a_{im})$, corresponds to a genotype. Second, we sum over every genotype relationship graph, $\boldsymbol{g}$, in graph space $\boldsymbol{\mathcal{G}}$ (equation (2)). The allelic data at different markers are assumed independent given the relationship graph. Third, for $j = 1, \dots, m$, it sums over every identity-by-descent (IBD) partition, $\boldsymbol{p}$, in partition space $\boldsymbol{\mathcal{P}}$ (equation (3)). The probability of the allelic data at a given marker conditional on a given IBD partition is a product over the cells, $\boldsymbol{c}$, in the partition (equation (4)), where $f(x)$ in equation (5) is the frequency of the allelic assignment x.

$$\mathbb{P}(\boldsymbol{y}|\boldsymbol{s}) = \sum_{\boldsymbol{a} \in \boldsymbol{\mathcal{A}}} \mathbb{P}(\boldsymbol{y}|\boldsymbol{a})\mathbb{P}(\boldsymbol{a}|\boldsymbol{s}), \text{ where } \mathbb{P}(\boldsymbol{y}|\boldsymbol{a}) = 1 \text{ by design.} \tag{1}$$

$$\mathbb{P}(\boldsymbol{a}|\boldsymbol{s}) = \sum_{\boldsymbol{g} \in \boldsymbol{\mathcal{G}}} \left(\prod_{j=1}^{m} \mathbb{P}(\boldsymbol{a}_{\cdot j}|\boldsymbol{g}) \right) \mathbb{P}(\boldsymbol{g}|\boldsymbol{s}), \text{ where } \mathbb{P}(\boldsymbol{g}|\boldsymbol{s}) = \begin{cases} 0 \text{ if } \boldsymbol{g} \text{ is incompatible with } \boldsymbol{s}, \\ {}^1/_{(\text{number of } \boldsymbol{g} \text{ compatible with } \boldsymbol{s})} \text{ otherwise.} \end{cases} \tag{2}$$

$$\mathbb{P}(\boldsymbol{a}_{\cdot j}|\boldsymbol{g}) = \sum_{\boldsymbol{p} \in \boldsymbol{\mathcal{P}}} \mathbb{P}(\boldsymbol{a}_{\cdot j}|\boldsymbol{p})\mathbb{P}(\boldsymbol{p}|\boldsymbol{g}), \text{ where } \mathbb{P}(\boldsymbol{p}|\boldsymbol{g}) = \begin{cases} 0 \text{ if } \boldsymbol{p} \text{ is incompatible with } \boldsymbol{g}, \\ {}^1/_{(\text{number of } \boldsymbol{p} \text{ compatible with } \boldsymbol{g})} \text{ otherwise.} \end{cases} \tag{3}$$

$$\mathbb{P}(\boldsymbol{a}_{\cdot j}|\boldsymbol{p}) = \prod_{\boldsymbol{c} \in \boldsymbol{p}} \mathbb{P}(\boldsymbol{a}_{\cdot j}|\boldsymbol{c}), \text{ where} \tag{4}$$

$$\mathbb{P}(\boldsymbol{a}_{\cdot j}|\boldsymbol{c}) = \begin{cases} f(\text{unique}(\boldsymbol{a}_{\boldsymbol{c}\, j})) \text{ if } |\text{unique}(\boldsymbol{a}_{\boldsymbol{c}\, j})| = 1, \\ 0 \text{ otherwise.} \end{cases} \tag{5}$$

where unique$(\boldsymbol{a}_{\boldsymbol{c}\, j})$ maps $\boldsymbol{a}_{\boldsymbol{c}\, j}$ (a subvector of $\boldsymbol{a}_{\cdot\, j}$) onto a set of alleles, and $|\text{unique}(\boldsymbol{a}_{\boldsymbol{c}\, j})|$ is the cardinality of the set. The numbers of $\boldsymbol{g}$ compatible with $\boldsymbol{s}$, and of $\boldsymbol{p}$ compatible with $\boldsymbol{g}$, are computed by enumerating all relationship graphs and IBD partitions, respectively. The total genotype count n is the sum of per-episode MOIs, n_t. The user can specify $n_t > \max(|\boldsymbol{y}_{t1}|, \dots, |\boldsymbol{y}_{tm}|)$, where $|\boldsymbol{y}_{tj}|$ is the number of different alleles observed in the tth infection at the jth marker for $j = 1, \dots, m$; otherwise n_t is set equal to $\max(|\boldsymbol{y}_{t1}|, \dots, |\boldsymbol{y}_{tm}|)$ internally.

k	Number of recurrent vivax malaria episodes.
m	Number of markers genotyped.
n	Number of parasite genotypes within and across episodes.
$t = 0, \ldots, k$	Index over episodes: $t = 0$ is the enrolment episode and $t > 0$ are recurrences.
$i = 1, \ldots, n$	Index over parasite genotypes.
$j = 1, \ldots, m$	Index over markers genotyped.
$\boldsymbol{s} = (s_1, \ldots, s_k)$	A 1-by-k vector of recurrent states in sequence space $\boldsymbol{\mathcal{S}}$, where $s_t \in \{C, L, I\}$ for $t = 1, \ldots, k$ recurrences and where C is a recrudescence, L is a relapse, and I is a reinfection.
$\boldsymbol{y} = (\boldsymbol{y}_{tj})$	A $(1+k)$-by-m matrix of sets of alleles observed in the tth infection at the jth marker.
$\boldsymbol{a} \in \boldsymbol{\mathcal{A}}$	A n-by-m matrix of phased alleles in matrix space $\boldsymbol{\mathcal{A}}$.
$\boldsymbol{g} \in \boldsymbol{\mathcal{G}}$	A graph in graph space $\boldsymbol{\mathcal{G}}$, whose vertices are parasite genotypes that are unlinked in the case of strangers; otherwise, they are linked by either clonal or sibling edges across infections, or by sibling edges within infections. Vertices $\boldsymbol{g}$ can also be expressed as a n-by-n matrix.
$\boldsymbol{p} \in \boldsymbol{\mathcal{P}}$	A partition in partition space, $\boldsymbol{\mathcal{P}}$, partitioning n genotypes into IBD clusters; $\boldsymbol{p}$ can also be expressed as a cluster graph or as an n-by-n matrix.
$\boldsymbol{c} \in \boldsymbol{p}$	An IBD cluster within an IBD partition (a cell in the partition $\boldsymbol{p}$).
$f(a_{ij})$	Frequency of the allele of the ith genotype at the jth marker in $\boldsymbol{a}$.

Table S2: Table of notation. Bold used to distinguish vectors, matrices, sets, and subsets from scalars.

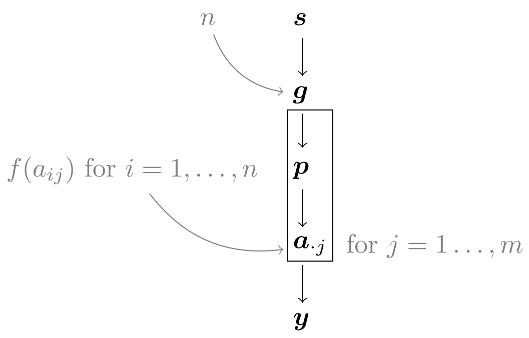

Figure S3: Pv3Rs model. Realised random variables in black; non-random, plug-in variables in gray.

2.3 Decomposing the phasing space

Because relationships are genotype-level attributes, in the description above, we first phase the data into genotypes and then sum over relationship graphs between genotypes. Consequently, the likelihood involves enumerating over the matrix space $\boldsymbol{\mathcal{A}}$ (equation (1)), whose cardinality grows exponentially with the number of markers. However, phasing does not limit the marker-scalability of the model in practice, because, assuming markers are conditionally independent given the relationship graph, we can decompose the phasing space such that the enumeration over allelic assignments is linear in the number of markers. Equations (1)–(2) can be expressed as

$$\mathbb{P}(\boldsymbol{y}|\boldsymbol{s}) = \sum_{\boldsymbol{a} \in \boldsymbol{\mathcal{A}}} \left(\mathbb{P}(\boldsymbol{y}|\boldsymbol{a}) \sum_{\boldsymbol{g} \in \boldsymbol{\mathcal{G}}} \mathbb{P}(\boldsymbol{a}|\boldsymbol{g}) \mathbb{P}(\boldsymbol{g}|\boldsymbol{s}) \right),$$

which we rearrange into

$$\mathbb{P}(\boldsymbol{y}|\boldsymbol{s}) = \sum_{\boldsymbol{g} \in \boldsymbol{\mathcal{G}}} \left(\mathbb{P}(\boldsymbol{g}|\boldsymbol{s}) \sum_{\boldsymbol{a} \in \boldsymbol{\mathcal{A}}} \mathbb{P}(\boldsymbol{y}|\boldsymbol{a}) \mathbb{P}(\boldsymbol{a}|\boldsymbol{g}) \right) = \sum_{\boldsymbol{g} \in \boldsymbol{\mathcal{G}}} \mathbb{P}(\boldsymbol{g}|\boldsymbol{s}) \mathbb{P}(\boldsymbol{y}|\boldsymbol{g})$$

by swapping the summations, where

$$\mathbb{P}(\boldsymbol{y}|\boldsymbol{g}) = \sum_{\boldsymbol{a} \in \boldsymbol{\mathcal{A}}} \mathbb{P}(\boldsymbol{y}|\boldsymbol{a}) \mathbb{P}(\boldsymbol{a}|\boldsymbol{g}) = \sum_{\boldsymbol{a} \in \boldsymbol{\mathcal{A}}} \prod_{j=1}^{m} \mathbb{P}(\boldsymbol{a}_{\cdot j}|\boldsymbol{g}).$$

In the last equality, we use the assumption of conditionally independent markers (equation (2)), and omit the term $\mathbb{P}(\boldsymbol{y}|\boldsymbol{a})$ as it is equal to 1 by design (equation (1)).

We now focus on how to rewrite $\mathbb{P}(\boldsymbol{y}|\boldsymbol{g})$ in a way that avoids explicitly enumerating over $\boldsymbol{\mathcal{A}}$. For each $j = 1, \ldots, m$, let $\boldsymbol{\mathcal{A}}_j$ be the set of possible values of $\boldsymbol{a}_{\cdot j}$ (alleles at marker j) consistent with the observed data $\mathbf{y}$. The key observation is that $\boldsymbol{\mathcal{A}}$ decomposes into a Cartesian product over $\boldsymbol{\mathcal{A}}_1, \ldots, \boldsymbol{\mathcal{A}}_m$. This implies that

$$\begin{aligned}\mathbb{P}(\boldsymbol{y}|\boldsymbol{g}) &= \sum_{\boldsymbol{a}\in\boldsymbol{\mathcal{A}}} \prod_{j=1}^{m} \mathbb{P}(\boldsymbol{a}_{\cdot j}|\boldsymbol{g}) \\ &= \sum_{\boldsymbol{a}_{\cdot 1}\in\boldsymbol{\mathcal{A}}_1} \cdots \sum_{\boldsymbol{a}_{\cdot m}\in\boldsymbol{\mathcal{A}}_m} \prod_{j=1}^{m} \mathbb{P}(\boldsymbol{a}_{\cdot j}|\boldsymbol{g}) \\ &= \prod_{j=1}^{m} \sum_{\boldsymbol{a}_{\cdot j}\in\boldsymbol{\mathcal{A}}_j} \mathbb{P}(\boldsymbol{a}_{\cdot j}|\boldsymbol{g}). \end{aligned} \tag{6}$$

Note that $\boldsymbol{\mathcal{A}}$ contains all allelic assignments, including those that are equivalent up to within-episode genotype permutations. If for each episode, we have a marker whose observed allelic diversity is equal to the MOI, we can exploit permutation symmetry to compute $\mathbb{P}(\boldsymbol{y}|\boldsymbol{s})$ more efficiently. Formally, let $\mathcal{Q}$ be the group of within-episode genotype permutations (remembering that a permutation is a function). Let $\boldsymbol{\mathcal{A}}^*$ be a subset of $\boldsymbol{\mathcal{A}}$ consisting of one representative allelic assignment from each equivalence class, as induced by within-episode genotype permutations. In other words, each equivalence class can then be expressed as $\{Q(\boldsymbol{a}^*)\}_{Q\in\mathcal{Q}}$ for some $\boldsymbol{a}^* \in \boldsymbol{\mathcal{A}}^*$. The assumption that for each episode, there is a marker whose observed allelic diversity matches the MOI implies that for each $\boldsymbol{a} \in \boldsymbol{\mathcal{A}}$, there is a unique $\boldsymbol{a}^* \in \boldsymbol{\mathcal{A}}^*$ and $Q \in \mathcal{Q}$ such that $\boldsymbol{a} = Q(\boldsymbol{a}^*)$. It follows that

$$\begin{aligned}\mathbb{P}(\boldsymbol{y}|\boldsymbol{s}) &= \sum_{\boldsymbol{g}\in\boldsymbol{\mathcal{G}}} \left(\mathbb{P}(\boldsymbol{g}|\boldsymbol{s}) \sum_{\boldsymbol{a}\in\boldsymbol{\mathcal{A}}} \mathbb{P}(\boldsymbol{a}|\boldsymbol{g}) \right) \\ &= \sum_{Q\in\mathcal{Q}} \sum_{\boldsymbol{a}^*\in\boldsymbol{\mathcal{A}}^*} \sum_{\boldsymbol{g}\in\boldsymbol{\mathcal{G}}} \mathbb{P}(Q(\boldsymbol{a}^*)|\boldsymbol{g})\mathbb{P}(\boldsymbol{g}|\boldsymbol{s}) \\ &= \sum_{Q\in\mathcal{Q}} \sum_{\boldsymbol{a}^*\in\boldsymbol{\mathcal{A}}^*} \sum_{\boldsymbol{g}\in\boldsymbol{\mathcal{G}}} \mathbb{P}(\boldsymbol{a}^*|Q^{-1}(\boldsymbol{g}))\mathbb{P}(\boldsymbol{g}|\boldsymbol{s}) \\ &= \sum_{Q\in\mathcal{Q}} \sum_{\boldsymbol{a}^*\in\boldsymbol{\mathcal{A}}^*} \sum_{\boldsymbol{g}\in\boldsymbol{\mathcal{G}}} \mathbb{P}(\boldsymbol{a}^*|Q^{-1}(\boldsymbol{g}))\mathbb{P}(Q^{-1}(\boldsymbol{g})|\boldsymbol{s}) \\ &= \sum_{Q\in\mathcal{Q}} \sum_{\boldsymbol{a}^*\in\boldsymbol{\mathcal{A}}^*} \sum_{\boldsymbol{g}\in\boldsymbol{\mathcal{G}}} \mathbb{P}(\boldsymbol{a}^*|\boldsymbol{g})\mathbb{P}(\boldsymbol{g}|\boldsymbol{s}) \\ &= |\mathcal{Q}| \sum_{\boldsymbol{g}\in\boldsymbol{\mathcal{G}}} \left(\mathbb{P}(\boldsymbol{g}|\boldsymbol{s}) \sum_{\boldsymbol{a}^*\in\boldsymbol{\mathcal{A}}^*} \mathbb{P}(\boldsymbol{a}^*|\boldsymbol{g}) \right), \end{aligned} \tag{7}$$

where Q^{-1} denotes the inverse of Q. The second equality appeals to the assumption that for each $\boldsymbol{a} \in \boldsymbol{\mathcal{A}}$, there is a unique $\boldsymbol{a}^* \in \boldsymbol{\mathcal{A}}^*$ and $Q \in \mathcal{Q}$ such that $\boldsymbol{a} = Q(\boldsymbol{a}^*)$. The third equality holds because $\mathbb{P}(\boldsymbol{a}|\boldsymbol{g})$ is invariant when the same within-episode genotype permutation is applied to $\boldsymbol{a}$ and $\boldsymbol{g}$. The fourth equality holds because $\mathbb{P}(\boldsymbol{g}|\boldsymbol{s})$ is invariant under any within-episode genotype permutation. The fifth equality holds because every within-episode genotype permutation maps the set of relationship graphs to itself (every $Q \in \mathcal{Q}$ is an automorphism on $\boldsymbol{\mathcal{G}}$). The final summation is more efficient to compute than the first by a factor of $|\mathcal{Q}|$.

The factorisation trick in (6) applies if $\boldsymbol{\mathcal{A}}^*$ can be written as a Cartesian product over markers. Practically, this can be achieved by the following construction: For each episode, identify a marker whose allelic diversity matches the MOI, and fix an allelic assignment of this marker for the genotypes belonging to this episode. For each marker j, let $\boldsymbol{\mathcal{A}}^*_j$ be the set of possible values of $\boldsymbol{a}_{\cdot j}$ for marker j that respects the aforementioned fixed allelic assignments (remembering that not all episodes whose alleles are fixed need be fixed at the same marker). We then define $\boldsymbol{\mathcal{A}}^*$ as the Cartesian product $\boldsymbol{\mathcal{A}}^*_1 \times \cdots \boldsymbol{\mathcal{A}}^*_m$. We illustrate this with an example where two alleles are observed at two of three markers genotyped in an enrolment infection, and the same scenario repeats for a recurrent infection, where one of the two markers are different:

$$\boldsymbol{y} = \begin{matrix} & j=1 & j=2 & j=3 & \\ \Bigg(& \{A,T\} & \{T\} & \{C,G\} & \Bigg) & t=0 \\ & \{A,T\} & \{T,C\} & \{G\} & & t=1 \end{matrix} . \tag{8}$$

We assume the most parsimonious explanation of the data: the number of genotypes in the first and second infections are both two. There are $|\boldsymbol{\mathcal{A}}_1| = 4$, $|\boldsymbol{\mathcal{A}}_2| = 2$, $|\boldsymbol{\mathcal{A}}_3| = 2$ ways to assign alleles for markers 1, 2, 3, respectively, leading to a total of $|\boldsymbol{\mathcal{A}}| = 16$ allelic assignments. We can construct $\boldsymbol{\mathcal{A}}^*$ by fixing allelic assignment for either marker $j = 1$ or marker $j = 3$ during episode 0, and fixing allelic assignment for either marker $j = 1$

or marker $j = 2$ during episode 1. Here, we fix an allelic assignment for marker $j = 1$ during each of the two episodes:

$$\boldsymbol{a}^*_{\mathrm{I}} = \begin{array}{c} \\ \left(\begin{array}{ccc} A & T & C \\ T & T & G \\ A & T & G \\ T & C & G \end{array}\right) \end{array}\begin{array}{l} \\ i=1,\ t=0 \\ i=2,\ t=0 \\ i=3,\ t=1 \\ i=4,\ t=1 \end{array}, \quad \boldsymbol{a}^*_{\mathrm{II}} = \left(\begin{array}{ccc} A & T & C \\ T & T & G \\ A & C & G \\ T & T & G \end{array}\right)\begin{array}{l} i=1,\ t=0 \\ i=2,\ t=0 \\ i=3,\ t=1 \\ i=4,\ t=1 \end{array},$$

(columns $j=1$, $j=2$, $j=3$)

$$\boldsymbol{a}^*_{\mathrm{III}} = \left(\begin{array}{ccc} A & T & G \\ T & T & C \\ A & T & G \\ T & C & G \end{array}\right)\begin{array}{l} i=1,\ t=0 \\ i=2,\ t=0 \\ i=3,\ t=1 \\ i=4,\ t=1 \end{array}, \quad \boldsymbol{a}^*_{\mathrm{IV}} = \left(\begin{array}{ccc} A & T & G \\ T & T & C \\ A & C & G \\ T & T & G \end{array}\right)\begin{array}{l} i=1,\ t=0 \\ i=2,\ t=0 \\ i=3,\ t=1 \\ i=4,\ t=1 \end{array}.$$

(columns $j=1$, $j=2$, $j=3$)

Each of the 16 allelic assignments is equivalent to exactly one of the assignments $\{\boldsymbol{a}^*_{\mathrm{I}}, \boldsymbol{a}^*_{\mathrm{II}}, \boldsymbol{a}^*_{\mathrm{III}}, \boldsymbol{a}^*_{\mathrm{IV}}\}$. In other words, $\boldsymbol{\mathcal{A}}$ can be partitioned into 4 equivalence classes of 4 allelic assignments each, where $\{\boldsymbol{a}^*_{\mathrm{I}}, \boldsymbol{a}^*_{\mathrm{II}}, \boldsymbol{a}^*_{\mathrm{III}}, \boldsymbol{a}^*_{\mathrm{IV}}\}$ are representatives of the 4 equivalence classes. To see how the factorisation (6) applies here, note that we have

$$\boldsymbol{\mathcal{A}}^* = \boldsymbol{\mathcal{A}}^*_1 \times \boldsymbol{\mathcal{A}}^*_2 \times \boldsymbol{\mathcal{A}}^*_3 = \{\boldsymbol{a}^{*1}_{\cdot 1}\} \times \{\boldsymbol{a}^{*1}_{\cdot 2}, \boldsymbol{a}^{*2}_{\cdot 2}\} \times \{\boldsymbol{a}^{*1}_{\cdot 3}, \boldsymbol{a}^{*2}_{\cdot 3}\},$$

where

$$\boldsymbol{a}^{*1}_{\cdot 1} = \overset{j=1}{\left(\begin{array}{c} A \\ T \\ A \\ T \end{array}\right)}\begin{array}{l} i=1,\ t=0 \\ i=2,\ t=0 \\ i=3,\ t=1 \\ i=4,\ t=1 \end{array},$$

$$\boldsymbol{a}^{*1}_{\cdot 2} = \overset{j=2}{\left(\begin{array}{c} T \\ T \\ T \\ C \end{array}\right)}\begin{array}{l} i=1,\ t=0 \\ i=2,\ t=0 \\ i=3,\ t=1 \\ i=4,\ t=1 \end{array}, \quad \boldsymbol{a}^{*2}_{\cdot 2} = \overset{j=2}{\left(\begin{array}{c} T \\ T \\ C \\ T \end{array}\right)}\begin{array}{l} i=1,\ t=0 \\ i=2,\ t=0 \\ i=3,\ t=1 \\ i=4,\ t=1 \end{array},$$

$$\boldsymbol{a}^{*1}_{\cdot 3} = \overset{j=3}{\left(\begin{array}{c} C \\ G \\ G \\ G \end{array}\right)}\begin{array}{l} i=1,\ t=0 \\ i=2,\ t=0 \\ i=3,\ t=1 \\ i=4,\ t=1 \end{array}, \quad \boldsymbol{a}^{*2}_{\cdot 3} = \overset{j=3}{\left(\begin{array}{c} G \\ C \\ G \\ G \end{array}\right)}\begin{array}{l} i=1,\ t=0 \\ i=2,\ t=0 \\ i=3,\ t=1 \\ i=4,\ t=1 \end{array},$$

The summation $\sum_{\boldsymbol{a}^* \in \boldsymbol{\mathcal{A}}^*} \mathbb{P}(\boldsymbol{a}^*|\boldsymbol{g})$ in (7) can be factorised as

$$\begin{aligned}
\sum_{\boldsymbol{a}^* \in \boldsymbol{\mathcal{A}}^*} \mathbb{P}(\boldsymbol{a}^*|\boldsymbol{g}) &= \mathbb{P}(\boldsymbol{a}^*_{\mathrm{I}}|\boldsymbol{g}) + \mathbb{P}(\boldsymbol{a}^*_{\mathrm{II}}|\boldsymbol{g}) + \mathbb{P}(\boldsymbol{a}^*_{\mathrm{III}}|\boldsymbol{g}) + \mathbb{P}(\boldsymbol{a}^*_{\mathrm{IV}}|\boldsymbol{g}) \\
&= \mathbb{P}(\boldsymbol{a}^*_{\mathrm{I}\cdot 1}|\boldsymbol{g})\mathbb{P}(\boldsymbol{a}^*_{\mathrm{I}\cdot 2}|\boldsymbol{g})\mathbb{P}(\boldsymbol{a}^*_{\mathrm{I}\cdot 3}|\boldsymbol{g}) + \mathbb{P}(\boldsymbol{a}^*_{\mathrm{II}\cdot 1}|\boldsymbol{g})\mathbb{P}(\boldsymbol{a}^*_{\mathrm{II}\cdot 2}|\boldsymbol{g})\mathbb{P}(\boldsymbol{a}^*_{\mathrm{II}\cdot 3}|\boldsymbol{g}) \\
&\quad + \mathbb{P}(\boldsymbol{a}^*_{\mathrm{III}\cdot 1}|\boldsymbol{g})\mathbb{P}(\boldsymbol{a}^*_{\mathrm{III}\cdot 2}|\boldsymbol{g})\mathbb{P}(\boldsymbol{a}^*_{\mathrm{III}\cdot 3}|\boldsymbol{g}) + \mathbb{P}(\boldsymbol{a}^*_{\mathrm{IV}\cdot 1}|\boldsymbol{g})\mathbb{P}(\boldsymbol{a}^*_{\mathrm{IV}\cdot 2}|\boldsymbol{g})\mathbb{P}(\boldsymbol{a}^*_{\mathrm{IV}\cdot 3}|\boldsymbol{g}) \\
&= \mathbb{P}(\boldsymbol{a}^{*1}_{\cdot 1}|\boldsymbol{g})\mathbb{P}(\boldsymbol{a}^{*1}_{\cdot 2}|\boldsymbol{g})\mathbb{P}(\boldsymbol{a}^{*1}_{\cdot 3}|\boldsymbol{g}) + \mathbb{P}(\boldsymbol{a}^{*1}_{\cdot 1}|\boldsymbol{g})\mathbb{P}(\boldsymbol{a}^{*2}_{\cdot 2}|\boldsymbol{g})\mathbb{P}(\boldsymbol{a}^{*1}_{\cdot 3}|\boldsymbol{g}) \\
&\quad + \mathbb{P}(\boldsymbol{a}^{*1}_{\cdot 1}|\boldsymbol{g})\mathbb{P}(\boldsymbol{a}^{*1}_{\cdot 2}|\boldsymbol{g})\mathbb{P}(\boldsymbol{a}^{*2}_{\cdot 3}|\boldsymbol{g}) + \mathbb{P}(\boldsymbol{a}^{*1}_{\cdot 1}|\boldsymbol{g})\mathbb{P}(\boldsymbol{a}^{*2}_{\cdot 2}|\boldsymbol{g})\mathbb{P}(\boldsymbol{a}^{*2}_{\cdot 3}|\boldsymbol{g}) \\
&= \mathbb{P}(\boldsymbol{a}^{*1}_{\cdot 1}|\boldsymbol{g})\left[\mathbb{P}(\boldsymbol{a}^{*1}_{\cdot 2}|\boldsymbol{g}) + \mathbb{P}(\boldsymbol{a}^{*2}_{\cdot 2}|\boldsymbol{g})\right]\left[\mathbb{P}(\boldsymbol{a}^{*1}_{\cdot 3}|\boldsymbol{g}) + \mathbb{P}(\boldsymbol{a}^{*2}_{\cdot 3}|\boldsymbol{g})\right] \\
&= \prod_{j=1}^{3} \sum_{\boldsymbol{a}^*_{\cdot j} \in \boldsymbol{\mathcal{A}}^*_j} \mathbb{P}(\boldsymbol{a}^*_{\cdot j}|\boldsymbol{g})
\end{aligned}$$

in the spirit of (6).

3 Additional details

The Pv3Rs differs to its prototype (Taylor et al., 2019) in several ways described in more detail below:

- The implementation of the Pv3Rs model scales to more markers, it is not limited to at most two recurrences per study participant, and it can handle a larger number of genotypes within and across episodes
- The Pv3Rs model does not contain a population-relatedness fudge factor
- A mathematically convenient assumption that lead to unfounded over-counting of allele frequencies in the prototype is replaced by summation over IBD partitions in the Pv3Rs model
- The Pv3Rs model assumption that at most two IBD clusters are compatible within a sibling cluster with a relationship graph renders Pv3Rs more liable to half-sibling misspecification

We also discuss below some challenges shared by both Pv3Rs and its prototype in regards to the relationship graph prior, and provide some commentary on Pv3Rs' limitations.

3.1 Computational scaling

The implementation of the prototype aimed to explicitly sum over all ways to phase the observed allelic data and thus was not scalable to more than nine markers. The implementation of the Pv3Rs model exploits the fact that phasing space can be decomposed such that enumeration over allelic assignments is linear in the number of markers (Section 2.3) and is thus scalable to many markers.

Moreover, Pv3Rs' enumeration of graphs is more efficient than its prototype. The prototype enumerates all graphs ignoring the transitivity requirement (see assumption 6), and subsequently decides which graphs are valid by checking for transitivity. Meanwhile, Pv3Rs avoids transitivity checks and directly enumerates over valid graphs by expressing graphs as nested partitions (see Pv3Rs Understand graph and partition enumeration vignette). Because the implementation of the Pv3Rs model is more efficient, it can sum over a larger graph space. As such it is not limited to at most two recurrences per study participant, and it can handle a larger number of genotypes within and across episodes.

The logarithm of the number of valid relationship graphs scales as $O(n \log n)$ (see the Benchmarking Pv3Rs website article), where n is the number of vertices (total MOI). This puts a practical limit on how many vertices Pv3Rs can handle. From experience, at 8 vertices, posterior computation takes about 5 minutes per marker (recall that computation time scales linearly with the number of markers). We expect this duration (and the memory cost) to increase by one order of magnitude for each additional vertex. Therefore, we do not recommend using Pv3Rs for total MOI larger than 8.

The explicit enumeration of relationship graphs is a fundamental limitation to the scalability of Pv3Rs (and its prototype). To make Pv3Rs scale to more genotypes, explicit enumeration could be replaced with Monte Carlo-based marginalisation using, e.g., a Markov chain Monte Carlo (MCMC) sampler. However, doing so requires building a generative model over graphs (which is beyond our current scope): if a uniform prior over graphs is used, calculating $\mathbb{P}(\boldsymbol{g}|\boldsymbol{s})$ for one specific $\boldsymbol{g}$ requires enumerating all relationship graphs compatible with $\boldsymbol{s}$. This negates the benefits of a potential MCMC approach, as calculating the acceptance probability of, say, a newly proposed $\boldsymbol{s}$ requires graph enumeration.

3.2 Deviation of Pv3Rs model assumptions from prototype

The prototype features a fudge factor $\alpha \in [0, 0.5]$ which can be assigned a non-zero value if IBD analyses suggest the trial participants are infected with parasites drawn from a population with elevated relatedness on average. In the Pv3Rs model, we initially considered including the inbreeding coefficient $F \in [0, 1]$, which can be viewed as an inter-gamete correlation (Hill, 1996): in a panmitic population of haploid individuals with equifrequent but finite lineages, strangers have relatedness F, and regular siblings have relatedness $\frac{1}{2}(1 + F)$ (twice the kinship coefficient of equation 13 in Speed and Balding (2015)). However, we decided to not include F in the Pv3Rs model because the allele frequencies used to compute the likelihood (equation (5)) are typically estimated using samples drawn from enrolment episodes. Sample allele frequencies encode locus-by-locus the average population-level relatedness (Mehra et al., 2025). As such, the inclusion of F would overcompensate for population-level relatedness.

The prototype over-counts frequencies, with disproportionate effects on different relationships, due to a mathematically convenient independence assumption. For example, if a person has three infections (two recurrent) in each of which the same allele, a say, is detected at a single marker, then for $\alpha = 0$ (see above), the likelihood (under the prototype) of twice reinfection and of twice recrudescence is $f(a)^6$ and $f(a)^3$, respectively, where $f(a)$ is the allele frequency. However, if reinfection is considered equivalent to drawing independently at random from the population, and if recrudescence is considered cloning with probability one, the likelihood of twice reinfection and of twice recrudescence should be $f(a)^3$ and $f(a)$, respectively. Over-countings occurs because the prototype takes a product over edges of relationship graphs (equation 8 of the supplement of (Taylor et al., 2019)), which amounts to assuming edges are conditionally independent. However, IBD relationships are

transitive, and therefore dependent. The Pv3Rs model instead takes a product over cells of IBD partitions (equation (4)). For each cell, we count one allele frequency if all alleles belonging to the genotypes within the cell at the jth marker are identical, and zero otherwise (equation (5)).

When enumerating all IBD partitions, $\boldsymbol{p}$, compatible with a given relationship graph, $\boldsymbol{g}$, we assume only $\boldsymbol{p}$ with at most two cells per relationship-graph sibling cluster are viable. This translates biologically to assuming siblings can draw alleles from at most two distinct parental genotypes. This in turn renders Pv3Rs more liable to half-sibling misspecification than its prototype; see Section 3.3. Under the prototype, all siblings are $1/2$ related but there is no limit on the number of distinct parental alleles they can draw from (because of the edge independence assumption).

3.3 Half-sibling misspecification

Our assumptions about siblings are not compatible with half siblings (see assumption 6 of Section 2.1 and above). Specifically, we have assumed that alleles of siblings are drawn from at most two parents, and that sibling relationships are transitive. Both of these are not necessarily true of scenarios with half siblings. Here, we give an example of how this can impact Pv3Rs.

Suppose that we have three parental genotypes, A, B, C ingested by a mosquito. Without loss of generality, we label the alleles of each marker of parental genotype A as a, so $A = aa\cdots a$. Similarly, we have $B = bb\cdots b$ and $C = cc\cdots c$. Consider a person infected by this mosquito, and experiences a relapse episode after an initial episode. Suppose that during the initial episode, there are two genetically unique offspring O_1 and O_2 present in the blood, both derived from parental genotypes A and B; during the relapse episode, there is one genetically unique offspring O_3 present in the blood, derived from parental genotypes A and C.

The true relationship graph is the graph with sibling edges between all three pairs of offspring genotypes (O_1 and O_2 are regular or meiotic siblings; O_1/O_2 and O_3 are half siblings). However, if there is some marker for which the offspring O_1, O_2, O_3 carry the alleles a, b, c respectively, the likelihood of this all-sibling graph is zero under the Pv3Rs model. This is because we assume that alleles of siblings are drawn from at most two parents, so the model only supports up to two distinct alleles within a sibling cluster. But because of half siblings, there are in fact three parents involved. The likelihood of the all-sibling graph is more likely to be zero when there are data on more markers. The graph with the highest likelihood under the Pv3Rs model is instead a graph with only one sibling edge, since a graph with exactly two sibling edges does not respect transitivity.

When this occurs, would Pv3Rs output probable relapse? The answer depends on the frequencies of the alleles shared between offspring, as the model is torn between placing an intra-episode sibling edge (between O_1 and O_2) or an inter-episode sibling edge (between one of O_1/O_2 and O_3). Loosely speaking, if the alleles shared between offspring from different episodes is overall rarer than alleles shared between offspring from the same episode, then Pv3Rs is likely to output probable relapse. In general, the behaviour of Pv3Rs with respect to half-sibling misspecification can be difficult to predict when more complex relationship graphs are involved. We have explored this theoretically in the Pv3Rs Understand half-sibling misspecification vignette and using simulations, of which a summary can be found in the half siblings section of the Pv3Rs website article Understand posterior probabilities.

3.4 Influence of uniform distribution over graphs

The uniform distribution over graphs allows us to we derive some bounds on Pv3Rs posterior probabilities. In what follows, for simplicity, we will assume that there is only one recurrent episode, and specify a uniform prior over the states. Further investigation of the uniform graph prior can be found in the Pv3Rs Understand graph-prior ramifications vignette.

Let $\mathcal{G}_\text{C}, \mathcal{G}_\text{L}$, and $\mathcal{G}_\text{I}$ denote subsets of the graph space $\mathcal{G}$, containing the relationship graphs compatible with recrudescence, relapse, and reinfection respectively. Recalling that the prior over states is uniform, the posterior odds of relapse to recrudescence is given by

$$o_{\text{L:C}} \coloneqq \frac{\mathbb{P}(\boldsymbol{y}|\text{L})}{\mathbb{P}(\boldsymbol{y}|\text{C})} = \frac{\sum_{\boldsymbol{g}\in\mathcal{G}_\text{L}} \mathbb{P}(\boldsymbol{y}|\boldsymbol{g})\mathbb{P}(\boldsymbol{g}|\text{L})}{\sum_{\boldsymbol{g}\in\mathcal{G}_\text{C}} \mathbb{P}(\boldsymbol{y}|\boldsymbol{g})\mathbb{P}(\boldsymbol{g}|\text{C})} = \frac{|\mathcal{G}_\text{C}|}{|\mathcal{G}_\text{L}|} \frac{\sum_{\boldsymbol{g}\in\mathcal{G}_\text{L}} \mathbb{P}(\boldsymbol{y}|\boldsymbol{g})}{\sum_{\boldsymbol{g}\in\mathcal{G}_\text{C}} \mathbb{P}(\boldsymbol{y}|\boldsymbol{g})} = \frac{|\mathcal{G}_\text{C}|}{|\mathcal{G}_\text{L}|} \left(1 + \frac{\sum_{\boldsymbol{g}\in\mathcal{G}_\text{L}\setminus\mathcal{G}_\text{C}} \mathbb{P}(\boldsymbol{y}|\boldsymbol{g})}{\sum_{\boldsymbol{g}\in\mathcal{G}_\text{C}} \mathbb{P}(\boldsymbol{y}|\boldsymbol{g})}\right),$$

where $\mathcal{G}_\text{L} \setminus \mathcal{G}_\text{C}$ is the subset of graphs compatible with relapse but not recrudescence (graphs where genotypes from the recurrent episode are not all clones from the initial episode). It follows that $o_{\text{L:C}} \geq |\mathcal{G}_\text{C}|/|\mathcal{G}_\text{L}|$. Note that this inequality is close to equality when $\sum_{\boldsymbol{g}\in\mathcal{G}_\text{L}\setminus\mathcal{G}_\text{C}} \mathbb{P}(\boldsymbol{y}|\boldsymbol{g}) \ll \sum_{\boldsymbol{g}\in\mathcal{G}_\text{C}} \mathbb{P}(\boldsymbol{y}|\boldsymbol{g})$. In other words, the data strongly suggests that recurrent genotypes are clones from the initial episode.

Next, note that $\mathcal{G}_\text{I}$ is a subset of $\mathcal{G}_\text{L} \setminus \mathcal{G}_\text{C}$, since clones are not compatible with reinfections, and all graphs are compatible with relapse ($\mathcal{G} = \mathcal{G}_\text{L}$). It follows that if $\sum_{\boldsymbol{g}\in\mathcal{G}_\text{L}\setminus\mathcal{G}_\text{C}} \mathbb{P}(\boldsymbol{y}|\boldsymbol{g}) \ll \sum_{\boldsymbol{g}\in\mathcal{G}_\text{C}} \mathbb{P}(\boldsymbol{y}|\boldsymbol{g})$, then $\sum_{\boldsymbol{g}\in\mathcal{G}_\text{I}} \mathbb{P}(\boldsymbol{y}|\boldsymbol{g}) \ll \sum_{\boldsymbol{g}\in\mathcal{G}_\text{C}} \mathbb{P}(\boldsymbol{y}|\boldsymbol{g})$ as well. When this happens, the posterior odds of reinfection to recrudescence, denoted $o_{\text{I:C}}$, would be close to zero.

Putting this together, we have

$$\begin{aligned}\mathbb{P}(\mathrm{C}|\boldsymbol{y}) &= \frac{\mathbb{P}(\boldsymbol{y}|\mathrm{C})}{\mathbb{P}(\boldsymbol{y}|\mathrm{C}) + \mathbb{P}(\boldsymbol{y}|\mathrm{L}) + \mathbb{P}(\boldsymbol{y}|\mathrm{I})} \\ &= \frac{1}{1 + o_{\mathrm{L:C}} + o_{\mathrm{I:C}}} \\ &\leq \frac{1}{1 + |\mathcal{G}_{\mathrm{C}}|/|\mathcal{G}_{\mathrm{L}}| + 0} \\ &= \frac{|\mathcal{G}_{\mathrm{L}}|}{|\mathcal{G}_{\mathrm{L}}| + |\mathcal{G}_{\mathrm{C}}|}. \end{aligned} \tag{9}$$

This inequality is close to equality when the data strongly suggests that recurrent genotypes are clones from the initial episode, corresponding to the case where "data are not sufficiently informative to distinguish between recrudescence and relapse" in the main text. Because of the uniform prior over graphs, the posterior probability of recrudescence under this regime is sensitive to the relative proportion of relationship graphs that are compatible with recrudescence, which depends on the MOI of each episode.

Equation (9) implies that regardless of how much data is available, the posterior probability of recrudescence can never exceed the upper bound $|\mathcal{G}_{\mathrm{L}}|/(|\mathcal{G}_{\mathrm{L}}|+|\mathcal{G}_{\mathrm{C}}|)$. We emphasises that the posterior probability of the correct recurrence state does not converge to 1 as the number of markers increase. This is because relationship graphs that are compatible with recrudescence are also compatible with relapse. Similar observations also apply to reinfection in place of recrudescence.

3.5 Dealing with misspecification

The violation of some Pv3Rs model assumptions is beyond the user's control, e.g., half-siblings. This type of misspecification is best addressed using sensitivity analyses and by cross-comparing with alternative approaches. The violation of other assumptions (e.g., mutually exclusive recurrence states) depends somewhat on study design and thus is within the user's control. This type of misspecification requires the user to think carefully about how to inpret Pv3Rs output in light of the assumptions violated by the user's study design. For example, if Pv3Rs is knowingly fit to data from infections that persist untreated, a high relapse probability under Pv3Rs might suggest superinfection: an ongoing infection (incompatible with reinfection) plus additional parasites from a new mosquito (incompatible with recrudescence).

Some assumptions have ramifications linked to transmission intensity (Table S3). For example, in low transmission settings, the uniform assumption over graphs might lead to recrudescence overestimation; meanwhile, clonal propagation and serial co-transmission might lead to reinfection underestimation. In high transmission settings, half-sibling misspecification and the uniform assumption over graphs might lead to reinfection overestimation; meanwhile, diverse and low density infections might lead to recrudescence underestimation.

Some model misspecifications may affect only a small proportion of all observed markers, e.g., genotyping errors, undetected genotypes due to low clone density, and *de novo* mutations. Such misspecifications can lead Pv3Rs to incorrectly eliminate the possibility of recrudescence, as recrudescence strictly requires all alleles observed from a recrudescence episode to also be observed from the preceding episode. As a result, when Pv3Rs outputs near-certain relapse, it may correspond to a genuine relapse, or a recrudescence that is mistaken as relapse due to e.g., genotyping errors. (Note that this becomes a non-issue in applications where relapse and recrudescence need not be distinguished, e.g. radical cure efficacy.) To distinguish between these two cases, we recommend using the proportion of markers incompatible with recrudescence as a diagnostic — in the case of genuine recrudescence, the proportion of markers incompatible with recrudescence would be close to the expected error rate; in the case of genuine relapse, this proportion would be higher. We provide more details on this issue in the Pv3Rs website article Understand genotyping errors. The simulation study investigated in the article is non-exhaustive; further development on the diagnosis of model misspecifications is merited.

References

Hill, W. G. (1996). Sewall wright's "systems of mating". *Genetics*, 143(4):1499.

Koella, J. C., SÖrensen, F. L., and Anderson, R. (1998). The malaria parasite, plasmodium falciparum, increases the frequency of multiple feeding of its mosquito vector, anopheles gambiae. *Proceedings of the Royal Society of London. Series B: Biological Sciences*, 265(1398):763–768.

Mehra, S., Neafsey, D. E., White, M., and Taylor, A. R. (2025). Systematic bias in malaria parasite relatedness estimation. *G3: Genes, Genomes, Genetics*, page jkaf018.

Assumption	Ramification whose violation frequency relates to transmission	Relationship with transmission
Mutually exclusive recurrence states	Eliminated probabilities of recrudescence and underestimated probabilities reinfection	Superinfections, and thus overlapping recurrences states, are more likely at high transmission
Perfectly detected alleles	Eliminated probabilities of recrudescence when only some per-marker alleles are detected	Undetected alleles are likely more prevalent in high transmission settings due to more diverse and lower-density infections
Conditionally independent markers	Overconfident but not necessarily biased inference	Inter-marker dependence is likely more prevalent in low transmission settings where linkage disequilibrium prevails
All siblings are regular siblings	Downwardly biased relapse probabilities due to half-sibling misspecification	Half-sibling misspecification is more likely in high transmission settings because the generation of half siblings requires mosquitoes to imbibe at least three genetically distinct parasite genotypes
Unstructured parasite population	Underestimated reinfection probabilities due to clonal propagation and serial co-transmission	Clonal propagation and serial co-transmission are more prevalent in low transmission settings
Conditionally uniform relationship graphs	Overestimation of probable recrudescence and probable reinfection	If clonal relapses are more likely in low transmission settings and if diverse hypnozoites are more like to accumulate in high transmission settings, the overestimation of probable recrudescence is likely in low transmission and the overestimation of probable reinfection is likely in high transmission

Table S3: A list of assumptions with ramifications linked to transmission intensity.

Scott, T. W. and Takken, W. (2012). Feeding strategies of anthropophilic mosquitoes result in increased risk of pathogen transmission. *Trends in parasitology*, 28(3):114–121.

Smith, R. C. and Barillas-Mury, C. (2016). Plasmodium oocysts: overlooked targets of mosquito immunity. *Trends in parasitology*, 32(12):979–990.

Speed, D. and Balding, D. J. (2015). Relatedness in the post-genomic era: is it still useful? *Nature Reviews Genetics*, 16(1):33–44.

Taylor, A. R., Foo, Y. S., and White, M. T. (2022). Plasmodium vivax relapse, reinfection and recrudescence estimation using genetic data. medRxiv preprint at https://doi.org/10.1101/2022.11.23.22282669.

Taylor, A. R., Watson, J. A., Chu, C. S., Puaprasert, K., Duanguppama, J., Day, N. P., Nosten, F., Neafsey, D. E., Buckee, C. O., Imwong, M., et al. (2019). Resolving the cause of recurrent plasmodium vivax malaria probabilistically. *Nature communications*, 10(1):1–11.

Wong, W., Wenger, E. A., Hartl, D. L., and Wirth, D. F. (2018). Modeling the genetic relatedness of plasmodium falciparum parasites following meiotic recombination and cotransmission. *PLOS Computational Biology*, 14(1):e1005923.
